# The Value of the Little Hospitals

**Published:** 1921-04-02

**Authors:** 


### The Value of the Little Hospitals.

It is often declared that the smaller hospitals will have to close their doors when the General Nursing Council brings out its rules. But the pessimists forget that a large number of institutions long ago decided for themselves that they were unable to grant a full certificate, and yet carry on very well. This is conspicuously the case in certain hospitals on the South Coast whose size does not admit of their running a training-school. Neither at the Hove Hospital nor at the Worthing Hospital, with its thirty-three beds, has there ever been any attempt to turn out fully-trained nurses. But both institutions did good service in giving a two-year course to probationers, avowedly as a preliminary to full general training. Until the late unhappy shortage of candidates set in they were never at a loss for young women glad to avail themselves of this introduction to nursing, and there can be no doubt that timid probationers would ultimately do better and be spared some adversity if they could first get acclimatised, as it were, to nursing conditions amid the pleasant surroundings of a small country-town hospital. The class of probationer attracted to this work did not always proceed to take further training. Very frequently it appears they discovered their real vocation to be matrimony, and it is to be believed that a two years' course of nursing work is one of the soundest forms of preparation for the mothers of the new generation. A good proportion of the probationers, however, went on to take complete general training.

The work at the Worthing Hospital is facilitated by the fact that all the wards are on the ground floor. The cases received are principally surgical. The main wards—men's, women's, and children's—are well planned and cheerful. There is an admirable operating-room with *x*-ray installation and a large out-patient and V.D. department, connected by a covered way with the hospital. At present no pay system has been introduced, though patients who can afford to do so are, of course, encouraged to make a donation. Worthing is in rather a peculiar position as regards paying patients, for there is a wealth of nursing homes within a short distance of the hospital, some adapted to all but the slenderest purses, and the need for pay beds is therefore less pressing than in some other localities.

The Matron of the Stratford-on-Avon Hospital, which stands much on the same footing, has appealed to the General Nursing Council for advice in the new position in which little hospitals will find themselves under registration, and it is probable their course of preliminary training will have to be standardised and brought into line. Whether they will be able to maintain a two-year course is at least doubtful, but the one-year probationer is not in great favour either in large or small institutions.

